# Investigating the Effects of Inhibition Training on Attentional Bias Change: A Simple Bayesian Approach

**DOI:** 10.3389/fpsyg.2018.02782

**Published:** 2019-01-21

**Authors:** Sandersan Onie, Lies Notebaert, Patrick Clarke, Steven B. Most

**Affiliations:** ^1^School of Psychology, UNSW Sydney, Sydney, NSW, Australia; ^2^School of Psychological Science, University of Western Australia, Perth, WA, Australia; ^3^School of Psychological Science, Curtin University, Perth, WA, Australia

**Keywords:** attentional bias, attention bias modification, inhibition control, dot probe, Bayesian statistics

## Abstract

Attention bias modification (ABM), in which participants are trained to direct attention away from negative information, has been shown to reduce anxiety. However, such findings have been inconsistent. Changes in attentional bias are often absent, suggesting need for further investigation of the underlying mechanisms of ABM, as well as better statistical methods to analyze ABM data in order to reduce inferential error. In this study, we (a) compared inhibition control training to standard ABM training conditions, and (b) demonstrated the benefits of using simple Bayesian analyses to analyze ABM data. We recruited 116 participants and assessed their attentional bias prior to and after training, which involved practice avoiding negative stimuli, attending to negative stimuli, or avoiding a non-emotional, exogenous attentional cue (inhibitory control training). Our results suggested no impact of any of the training conditions on attentional bias. We further demonstrate Bayesian analyses may help control for both Type I and Type II error relative to a frequentist approach.

## Introduction

The world provides stimulation beyond what we can process at any one time, and attention mechanisms help us winnow this richness down to the select chunks of information that make up our lived experience. We are not always in control of what those chunks are; some stimuli capture attention because of their featural salience (Theeuwes, 1992), and some stimuli capture attention because of their negative emotional meaning (MacLeod et al., 1986; Most et al., 2005; Onie and Most, 2017). When the latter occurs, this is known as a negative attentional bias, and extreme manifestations of this type of bias have been implicated in the development and maintenance of emotional disorders such as anxiety and depression (Eysenck, 1992; Van Bockstaele et al., 2014).

The most widely used task for measuring attentional bias is the Dot Probe (MacLeod et al., 1986). In a typical Dot Probe trial, a negative and a neutral stimulus (commonly words or images) are briefly presented at separate screen positions. The stimuli then disappear, with one of the items replaced by a probe to which the participant must respond. Response latencies are taken as an indication of attentional allocation: a consistently quicker response on trials where probes replaced the negative stimulus (congruent trials) than to trials where probes replaced the neutral stimulus (incongruent trials) suggests preferential attention to the location of negative stimuli (i.e., a negative attentional bias). Many studies have demonstrated that individuals high in anxiety vulnerability show an increased attentional bias to negative information relative to individuals low in anxiety vulnerability (Bar-Haim et al., 2007).

Past studies have suggested that training can reduce negative attentional biases, with a consequent impact on emotional vulnerability (Van Bockstaele et al., 2014). In training sessions, the original Dot Probe task (used primarily for assessment) is modified by introducing a contingency, such that probes consistently replace either the negative stimulus or the neutral stimulus in order to respectively, increase or attenuate attentional biases to negative stimuli (Mathews and MacLeod, 2002). This procedure has been implemented in clinical settings, and successful reduction in negative attentional bias has been linked with a reduction in anxiety symptomatology (MacLeod and Clarke, 2015; Price et al., 2016). However, a persistent concern within the Dot Probe ABM literature is that although studies have demonstrated a reduction in anxiety when attentional bias is successfully reduced, often this change in attentional bias is not achieved (e.g., Clarke et al., 2014; Notebaert et al., 2015). These findings underscore the need to better understand the specific mechanisms contributing to attentional change in order to improve training methods resulting in consistent outcomes.

One mechanism that may contribute to a negative attentional bias is an impairment in inhibitory control. For example, past studies have found that anxious individuals show a deficit in inhibitory control measured using the anti-saccade task (Derakshan et al., 2009; Wieser et al., 2009), a task which requires the individual to inhibit reflexive eye-movements toward a neutral stimulus on the screen. Specifically, Derakshan et al. (2009) showed that high anxious individuals were slower than low anxious individuals in the anti-saccade task, but showed no difference in a pro-saccade task, in which the cue and the target were in the same location. This suggests that anxious individuals did not differ in initiating attention to a neutral stimulus, but rather specifically in inhibiting the allocation of attention to a salient distractor. Models such as the attentional control theory (Eysenck et al., 2007; Eysenck and Derakshan, 2011) have postulated that anxiety-linked deficits in executive control, specifically deficits in the inhibitory function, may play a maintaining role in a negative attentional bias (see Heeren et al., 2013 for a review).

Therefore, it is possible that ABM procedures achieve their impact on attentional bias by training inhibitory control. Consistent with this notion, previous research has shown that individuals who were trained to attend away from threating stimuli (e.g., words reflecting social threat) showed improved performance in a post-training attentional control task (Chen et al., 2015), suggesting that ABM training improved inhibitory attentional control. There was no significant difference between trials using negative, neutral or positive stimuli in the attentional control assessment, suggesting that this increase in inhibitory control was not emotion-specific. Furthermore, an fMRI study showed that ABM increased neural activity in lateral frontal regions, areas found to play a role in inhibitory control (Browning et al., 2010), and another study found that by stimulating the dorsolateral prefrontal cortex (linked to attentional control) attentional bias was more readily modified (Clarke et al., 2014). Such findings are again consistent with the notion that one of the mechanisms modified by ABM may be general inhibitory control, and also consistent with recent suggestions that the attentional biases often observed among clinical populations actually reflect a more domain-general inefficiency of attentional control (McNally, 2018).

One potential way to test whether increasing general inhibitory attentional control contributes to reductions in negatively biased attention similar to ABM procedures is to adapt the anti-cueing task into a training task, similar in format to ABM tasks. The anti-cueing task is a behavioral adaptation of the anti-saccade task that uses response time as the dependent variable instead of eye-movements. In the anti-cueing task, a single pre-target cue appears, with the target then appearing in the opposite location (Posner et al., 1982; Cain et al., 2014). This assessment task can be adapted into a training task to reduce attentional deployment to a non-emotional, but salient, pre-potent stimulus. This might be achieved by presenting multiple trials in which only a single neutral pre-target stimulus appears, with the target then appearing in the opposite location. We hypothesize that by training the individual to consistently orient away from a neutral, pre-potent stimulus, it may be possible to train inhibitory control. Because this training condition would not include emotional stimuli, it may better target pure inhibitory control, without a potentially additional role of emotion regulation.

Thus, in the current study, we sought to compare the impact of inhibition control training to regular ABM training. Specifically, we assessed whether inhibitory control training produced a similar reduction in attentional bias as a standard avoid-negative condition of the dot probe task. If inhibitory control alone can contribute to attentional bias change in similar fashion as ABM, it is predicted that, relative to an attend-negative ABM condition (where no inhibitory control training is hypothesized to be involved), the inhibitory control training and avoid-negative training conditions should produce similar changes in attentional bias.

An additional aim of the present study was to include analyses to address a limitation of past studies. Specifically, inconsistent outcomes of ABM studies (i.e., observations either of significant change in attentional bias or of no significant change) may stem from lack of power in any given study. A lack of power or precision may lead to either (a) not enough power to detect a true change (Type-II error), or (b) a lack of power leading to spurious significant findings (Type-I error), potentially resulting in inaccurate conclusions in low-powered studies (Button et al., 2013). The low test-retest reliability of typical Dot Probe measures (Schmukle, 2005) may also contribute to the issue of power, as simulations demonstrate that low task reliability yields lower power (Kanyongo et al., 2007). Therefore, since low power may lead to inaccurate conclusions, better statistical methods are needed to determine whether the study is underpowered.

As part of this study, we compared the use of a Bayesian inference to a frequentist approach (the widely used approach using *p*-values for inference) in analyzing post-ABM attentional bias. Bayesian approaches are often capable of distinguishing between lack of power/precision and lack of an effect, and they have steadily garnered favor in many corners of the psychological literature (Andrews and Baguley, 2013). This approach starts with *a priori* beliefs (“priors”) about the direction or size of an effect, which are ultimately combined with observations (evidence) and data to yield updated beliefs (“posteriors”). This can be illustrated with an intuitive example: if you only have superficial familiarity with a colleague who always appears to be carefree, your impression of them might change drastically the first time you encounter them in a depressed state. In contrast, for those who know your colleague better and who have a more well-rounded understanding of them, witnessing the colleague’s depression might not change their impression as much. In short, what we take away from an event (posteriors) depends on our prior expectations (priors), even if the event itself is held consistent (evidence). It is worth noting that if all observers’ perceptions of your colleague were absolutely identical after the depressive episode, the observers would have identical posteriors of the event. However, in most cases, the resulting posteriors will be constrained by the initial priors. Ultimately the heart of Bayesian inference is combining priors with current evidence to yield posteriors.

Bayesian inference takes the form of model comparison, evaluating the degree to which the data support one model over the other (e.g., null vs. alternative), and asks the question: ‘how many times more likely is one model than the other?’ This is represented with a Bayes Factor, a numeric value that represents how likely the alternative (H1) is relative to the null (H0). For example, a Bayes factor of 10 suggests that the alternative is 10 times more likely than the null, a Bayes factor of 0.1 suggests that the null is 10 times more likely than the alternative, and a Bayes factor of 1 suggests the null and the alternative are equally likely, with insufficient evidence to suggest one direction over the other. This contrasts with typical *p*-values, which allow investigators to infer either that there is evidence to support a claim (*p* < 0.05) or that there is not enough evidence to support a claim (*p* > 0.05), and it is often unclear whether the latter outcome indicates support for the null or a lack of experimental- or statistical- power. In contrast, Bayes factors allow researchers to distinguish between three outcomes: support for the null, support for the alternative, or ambiguous results. This is a useful way to distinguish between the absence of an effect and absence of power. Note that, here, power is defined not as the probability of detecting an effect, but rather the precision of estimates stemming from the amount of evidence collected. This is an important feature, as low powered studies have been shown to lead to spurious findings and a lack of reproducibility (Button et al., 2013). It can be argued that this is a more objective measure of power than *a priori* power analyses, since power is directly driven by evidence in the present data rather than data collected in another study or meta analyses, which may be prone to publication bias.

In sum, this study had two main aims: to test whether inhibitory control training produced a similar reduction in attentional bias as a standard avoid-negative condition of ABM, and to demonstrate the benefits of using Bayesian inference in analyzing ABM data.

## Materials and Methods

### Participants

120 participants (41 males, 79 females, M_age_ = 22.37, *SD*_age_ = 4.23) from the general community were recruited through an online system (SONA) and were compensated AUD $15 for their time.

The sample size was determined *a priori*, drawing from between-subjects effect sizes from past studies (η^2^ = 0.06–0.15; e.g., Grafton et al., 2012; Clarke et al., 2014; Notebaert et al., 2015). Power analyses performed in Clarke et al. (2017), using a similar repeated measures design, suggested 105 participants would yield 80% power. This study was carried out in accordance with the recommendations of ‘the Human Research Ethics Advisory Panel at UNSW’ with written informed consent from all subjects. All subjects gave written informed consent in accordance with the Declaration of Helsinki. The protocol was approved by the Human Research Ethics Advisory Panel.

### Design

Participants completed a questionnaire indexing negative affect followed by a pre-training Dot Probe measure of attentional bias. Then, the participants were randomly allocated to one of three between-subjects training conditions based on arrival time to the lab (e.g., participants 1, 4, and 7 would be in the same training condition). One training condition presented negative-neutral stimulus pairs, combined with a contingency that encouraged participants to attend toward the negative stimulus (attend-negative condition). One presented negative-neutral stimulus pairs, combined with a contingency that encouraged participants to attend away from the negative stimulus (avoid-negative condition). A third presented a single non-emotional stimulus (rendering it salient), combined with a contingency that encouraged participants to attend away from it (inhibitory control training condition). All participants then engaged in a post-training Dot Probe measure of attentional bias.

The study took the form of a single session training study to assess the impact of inhibitory suppression on attentional bias.

### Materials

#### Hardware

Stimuli were presented on a 24-in. BenQ XL2420T LED monitor with 1920 × 1080 resolution and 120-Hz refresh rate. Head position was not fixed.

#### Questionnaires

The Depression, Anxiety, and Stress Scale 21 (DASS-21; Lovibond and Lovibond, 1995) was used to investigate whether there were any pre-existing differences in negative affect amongst the training groups. Participants had to indicate how much statements applied to them over the past week e.g., “I found it hard to wind down,” on a four-point scale ranging from ‘Did not apply to me at all – NEVER,’ to ‘Applied to me very much, or most of the time – ALMOST ALWAYS’. See Table 1 for cut-off scores.

**Table 1:** Cut-off scores for DASS-21, taken from Lovibond and Lovibond (1995).

	Depression	Anxiety	Stress
Normal	0–9	0–7	0–14
Mild	10–13	8–9	15–18
Moderate	14–20	10–14	19–25
Severe	21–27	15–19	26–33
Extremely severe	28+	20+	34+

#### Attentional Task Stimuli

For the negative-neutral stimulus pairs, 75 negative and 75 neutral images consisting of images of people and animals were taken from various sources, including web searches and the International Affective Picture System (Lang et al., 1997), all of which were then collectively rated by independent raters on Amazon’s Mechanical Turk. The negative images often depicted mutilated bodies and disgust images (e.g., soiled toilets). Neutral images often depicted people in everyday scenes as well as household objects. One-hundred and ninety individuals rated these images on two dimensions: valence and arousal.

To rate the valence, we asked the participants ‘How does this image make you feel?’ to which they answered on a scale ranging from -9 (extremely negative) to 9 (extremely positive). For arousal, we asked the participants ‘how intense is this image?’ to which they responded on a scale ranging from 0 (not at all intense), to 9 (extremely intense).

Likelihood ratio tests confirmed that negative images were rated more negatively than neutral images χ(1) = 14,459, *p* < 0.001, and that negative images were rated as more intense than neutral images χ(1) = 12,736, *p* < 0.001 (Valence: M_neg_ = -5.594, *SD*_neg_ = 1.1512, M_neut_ = 1.186, *SD*_neut_ = 0.853, Arousal: M_neg_ = 6.155, *SD*_neg_ = 1.168, M_neut_ = 1.887, *SD*_neut_ = 0.264).

For the inhibitory control training condition, images depicting landscapes – absent of depictions of people or animals – were used. We used photos of landscapes rather than simpler stimuli so that naturalistic scenes were employed across all training conditions.

The same stimulus set was used in the pre-training attentional bias assessment task and the attentional training task, using 50 of the 75 images from the neutral and negative image pool each (except for the inhibitory control training condition, which used only landscapes). Post-training assessment used a different stimulus set (the other 25 images from each of the neutral and negative image pools) to ensure any training effects were associated with the stimulus valence rather than the specific stimuli themselves.

#### Attentional Bias Assessment Task

During pre-training and post-training assessment participants were instructed to indicate the left/right direction of an arrow probe using directional keys, and to try ignore the images that would appear on the screen prior to the target arrow.

On each trial, a fixation cross appeared at the center of the screen for 100 ms, followed by two 11.5 cm × 8.5 cm images (a neutral and negative image) placed with their medial edges 7.5 cm above and below central fixation (15 cm from one image’s edge to another). We used a 500 ms exposure time for the images, consistent with past studies (Clarke et al., 2017). The images disappeared to reveal an arrow (3.5 cm × 3.5 cm) behind one of the images, which pointed either left or right and remained until participants made a response indicating the arrow’s direction. The two images were placed at the top and bottom of the screen rather than left or right as per recommendations to improve task reliability (Price et al., 2015).

For each assessment phase, participants completed 120 trials with a short break after 60 trials. Trial type (Congruent, in which the probe appeared behind the negative stimuli, vs. Incongruent, in which the probe appeared behind the non-negative stimuli) and Probe type (arrow pointing left or right) were equally and randomly allocated throughout. In pre-training, 50 negative and 50 neutral images were randomly distributed amongst 120 trials (each trial having 1 negative and 1 neutral image), resulting in each image being presented 2–3 times. In post-training, 25 negative and 25 neutral images were randomly distributed amongst 120 trials, resulting in each image being presented 4–5 times.

#### Training Task

During the training phase, participants completed 720 trials with a short break after every 80 trials.

Trials and instructions were identical to pre-training assessment except: In the attend-negative condition the probe was always behind the negative stimuli, and in the avoid-negative condition the probe was always behind the neutral stimuli. We adopted this contingency based on past studies (Grafton et al., 2014; Milkins et al., 2016; Clarke et al., 2017). This was to train attention toward and away from negative stimuli, respectively. In the inhibitory control training condition, only one pre-probe stimulus was present, and the probe always appeared in the other location.

**FIGURE 1:**
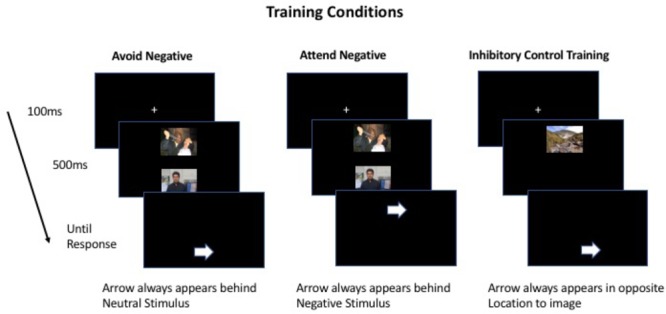
Schematic of training conditions. Image location (top and bottom), arrow location (top or bottom), as well as arrow direction (left or right) were randomized.

For avoid-negative and attend-negative training conditions, participants were trained on the 50 images used in the pre-training assessment randomly distributed amongst 720 trials. In the inhibitory control condition, 50 images depicting landscapes were used instead. A schematic of the different training conditions can be found in Figure 1.

### Procedure

Participants were tested in individual testing rooms. All tasks were completed on computers, starting with a demographics questionnaire and the DASS-21 on Qualtrics (a website based survey tool). Next, participants completed the pre-training attentional bias assessment, before proceeding with the attentional training task. They then finished with the post-training attentional bias assessment. Participants were debriefed at the end of the study.

### Data Availability

Pre-registration of the aims, methods, and analysis plan, data, and analysis output can be found at: https://osf.io/hfr9s/.

## Results

### Data Preparation

Accuracy for all participants and across congruency was high for pre- and post-training (M_Accuracy_ = 97.6%, *SD*_Accuracy_ = 1.5%). All participants met a pre-defined inclusion criterion of M_Accuracy_ > 80%.

Probe reaction times were prepared by removing all observations faster than 200 ms and slower than 2000 ms. On average, 2–3 trials were removed for each individual. Following that, reaction times outside three standard deviations from each participant’s own mean, separating pre and post training, congruent and incongruent trials to eliminate outliers. Attentional bias indices were then calculated separately for pre- and post- training: aggregate scores for congruent trials were subtracted from aggregate scores for incongruent trials. A positive attentional bias index indicates a quicker response in congruent trials relative to incongruent trials and suggests an attentional bias toward negative stimuli. Five participants were removed from analyses due to pre-training bias scores that were three standard deviations or more from the mean.

Note that past studies have combined pre and post attentional bias scores when eliminating outliers three standard deviations from an individual’s own mean (e.g., Clarke et al., 2017). Our pattern of results remains the same between both exclusion methods.

Past studies have criticized the use of aggregate bias scores, favoring other indices such as the bias variability score (Iacoviello et al., 2014). However, simulations have found that these indices can fluctuate without an actual attentional bias, and rather reflect response time variability (Kruijt et al., 2018). Also, despite past issues with reliability, aggregate bias indices have been shown to be reliably associated with anxiety (Bar-Haim et al., 2007).

Seven participants did not have DASS-21 data due to a system error in which their data were not recorded, and these participants were not included in analyses involving DASS-21. Overall, there were 40 participants in the avoid-negative condition, 37 in the attend-negative condition, and 38 in the inhibitory training condition.

### Attentional Bias Analyses

We first report data analyses using a frequentist method and follow this with a Bayesian analysis. In the interest of making these analyses accessible, both analyses were done in JASP, an open source software which includes both frequentist and Bayesian analyses. This point and click software was developed to introduce people to Bayesian analysis using a familiar interface.

#### Frequentist Analysis

First, we investigated whether there were any pre-existing differences between individuals in the attend-negative, avoid-negative, and inhibitory control conditions prior to training. We performed a one-way ANOVA on the pre-training attentional bias index, which showed no evidence for group differences, *F*(2,112) = 0.219, *p* = 0.804, η^2^ = 0.004.

Next, we investigated whether there were any pre-existing differences in negative affect between the different groups via a similar one-way ANOVA on the DASS total score, as well as the depression, anxiety, and stress subscales. The analysis revealed no significant difference between the groups on any of the scales or subscales, DASS: *F*(2,106) = 0.180, *p* = 0.835, η^2^ = 0.003; DASS-D: *F*(2,106) = 0.018, *p* = 0.982, η^2^ < 0.001; DASS-A: *F*(2,106) = 0.219, *p* = 0.804, η^2^ = 0.004; DASS-S: *F*(2,106) = 0.586, *p* = 0.558, η^2^ = 0.011. See Table 2 for means and standard deviations, and reliability indices.

**Table 2:** Means and standard deviations of the Depression, Anxiety, and Stress scale.

	DASS total	DASS-D	DASS-A	DASS-S
Mean	10.70	3.376	3.138	4.183
SD	8.919	3.310	3.081	3.675
Reliability	0.932 (0.928)	0.864 (0.853)	0.810 (0.794)	0.871 (0.869)

*Reliability indices are McDonald’s ω, with Cronbach’s α in the brackets.*

To test whether there was a significant difference between the impact of the three training conditions on attentional bias, we performed a repeated measures ANOVA with time (pre and post training) as a within subjects factor and training condition as a between subjects factor. The analysis yielded no main effect of time, *F*(1,112) = 0.160, *p* = 0.690, η^2^ = 0.001, nor a main effect of training condition, *F*(2,112) = 0.523, *p* = 0.594, η^2^ = 0.009, nor an interaction between time and condition *F*(2,112) = 1.151, *p* = 0.860, η^2^ = 0.003. Therefore, we failed to reject the null hypothesis that there was no impact of training on attentional bias. However, note that it is unclear from this analysis whether this reflects the absence of an effect or a lack of power to find an existing effect, as we cannot give support for the null using the frequentist analysis.

To ensure between-software consistency, the same analysis was performed in SPSS, with the exact same outcome. The output can be found in our osf page noted above. See Table 3 for attentional bias means and Table 4 for reliability values.

**Table 3:** Attentional Bias Indices for three training conditions.

Training conditions	Pre-Training AB index	Post-Training AB index	Attentional bias change mean
Avoid negative	1.256 (11.49)	–0.484 (13.62)	–1.740 (16.24)
Attend negative	–2.918 (8.915)	0.376 (12.20)	3.295 (16.22)
Inhibitory control	–0.623 (12.40)	0.575 (11.74)	1.204 (13.80)

*All indices are in ms. Standard deviations are in brackets. AB, attentional bias.*

**Table 4:** Reliability indices of Dot Probe scores.

Training condition	Pre-Training			Post-Training		
	Cong	Incong	AB index	Cong	Incong	AB index
Attend negative	0.81 (0.98)	0.78 (0.98)	0.51 (0.78)	0.59 (0.96)	0.81 (0.97)	0.53 (0.76)
Avoid negative				0.97 (0.99)	0.5 (0.99)	0.50 (0.81)
Inhibition				0.66 (0.95)	0.65 (0.96)	0.44 (0.69)

*Cong refers to the congruent trial type, Incong refers to the incongruent trial type. AB index reliability indices are computed by averaging across reliability indices obtained from differences scores from 100 different combinations of within participant incongruent – congruent scores. Values are McDonald’s ω, whilst values in brackets are Cronbach’s α.*

#### Bayesian Analysis

Although Bayes Factors are often reported as BF_10_, which refers to the probability of the alternative model against the null, here we report findings using BF_01_, which is the probability of the null relative to the alternative hypothesis (the inverse probability of BF_10_: BF_01_ = 1/BF_10_). This is to help interpretability of the Bayes factors for null findings; for example, ‘we are 15 times more likely to find the null’ is more intuitively interpretable than ‘we are 0.067 times more likely to see the alternative hypothesis’. We used Jeffrey’s scale to interpret Bayes factors (Jeffreys, 1961), which places labels on Bayes factors (e.g., BF = 1–3 is anecdotal evidence, BF = 3–10 is moderate evidence and BF = 10–30 is strong evidence). Whilst unlike the frequentist approach there are no strong cutoffs, a Bayes Factor of 10 is often used to indicate compelling evidence (Wagenmakers et al., 2015; Aczel et al., 2018). The following analyses were done using the Jeffreys-Zellner-Siow (JZS) default priors for ANOVA (Rouder and Morey, 2012). Note that the default priors selected in JASP has been shown to operate well with a wide range of ANOVA designs and provides a good balance between very strong to uncertain priors.

First, we investigated whether there were any pre-existing attentional bias differences between the groups prior to training. We performed a one-way ANOVA investigating group differences and found moderate evidence to suggest there were no pre-existing differences BF_01_ = 7.332.

Next, we investigated whether there were any pre-existing differences in negative affect between the different groups. We performed four one-way ANOVA to see whether different training groups differed in their DASS subscale or total score. The analysis revealed moderate to strong evidence that there were no pre-existing differences between the groups, DASS: BF_01_ = 10.063, DASS-D: BF_01_ = 11.494, DASS-A: BF_01_ = 9.745; DASS-S: BF_01_ = 7.238.

To test our main hypothesis, we performed a repeated measures ANOVA with time (pre and post training) as a within subjects factor and training condition as a between subjects factor. The analysis revealed moderate evidence suggesting there was no effect of time BF_01_ = 6.135, and strong evidence suggesting there was no effect of training condition BF_01_ = 10.638. In line with the model comparison nature of Bayesian statistics, to obtain the evidence for the interaction term we divided the Bayes Factor for the model containing the interaction term by the Bayes Factor for the model with only the main effects. The analysis yielded strong evidence suggesting there was no interaction BF01 = 10.417.

#### Insufficient Power May Lead to Inaccurate Conclusions

To illustrate the relative ability of Bayesian and frequentist approaches to distinguish lack of effect from lack of precision, we repeated both the frequentist and Bayesian analysis, and we intentionally reduced precision and power by reducing sample size to a sample commonly observed in early attentional bias modification research (Mathews and MacLeod, 2002). We repeated the above-described frequentist and Bayesian analysis 1000 times, testing for group differences in attentional bias change while restricting the analyses to 15 randomly selected samples (participants) in each condition. We then obtained a percentage of the results which were considered significant in the frequentist tradition (*p* < 0.05) and a percentage of the results that had strong evidence in the Bayesian framework (BF10 > 10). From the 1000 iterations of this analysis, we found that 1.6% of the frequentist analyses yielded significant results (Type I error) and 98.4% would have concluded that there was no effect (a potential Type II error due to low power), In contrast, using the Bayesian framework, 0.1% of the Bayes Factors suggested there was a strong effect, 0.1% suggested strong evidence for a lack of an effect, and 99.8% suggested there was only either anecdotal or moderate evidence in either direction.

## Discussion

In this study, we had two overarching aims: to investigate whether training participants to ignore pre-potent, non-emotional stimuli would yield similar results to training individuals to ignore negative stimuli, and to demonstrate the utility of using Bayesian analyses to analyze post-ABM attentional bias.

Our results suggest a single session of inhibition training (i.e., training participants to ignore pre-potent, non-emotional stimuli) did not shift attentional bias in healthy participants in either the Frequentist or the Bayesian analysis. However, there was also no significant bias change in either of the two other training conditions, limiting our ability to rule out the possibility that inhibition training had no impact similar to those frequently reported in the attentional bias modification literature. Notably, the absence of attentional bias change in the attend-negative and avoid-negative training conditions is consistent with a number of previous studies that have also failed to achieve significant changes in attention bias using tasks based on the traditional dot probe paradigm (Clarke et al., 2014, 2017; Everaert et al., 2015; Notebaert et al., 2015). This underscores the need to understand the processes via which bias change is achieved, and also to explore potentially alternative ways of changing attention bias.

There could be several potential reasons as to why there was no significant bias change. One potential reason there was no reduction of attentional bias in the ‘avoid negative’ condition is that we only used healthy participants without pre-screening for anxiety. It is also possible that multi-session, high dose retraining sessions are required to reliably modify attention. However, past studies have found success in modifying attentional biases within a single session in healthy participants (e.g., Chen et al., 2015). Therefore, we have reason to believe that rather than simply increasing the dose, we need to further investigate the underlying mechanisms for attentional bias change to achieve consistency in modifying attention. In addition, we sought to investigate whether inhibition training could account for some of the previously observed patterns in the avoid-negative condition, and other studies that have reported success using one session of ABM training have typically included fewer training trials than in the present study.

Other potential reasons may lie with our specific methodology. One possibility is that button press reaction times may be too crude of a measurement to assess the differences present in these biases. Instead, other implicit measures of attention such as eye tracking could be used. Another possibility is that inhibitory control training did not generate change due to using images depicting landscapes. We initially chose to use these images so that naturalistic scenes were used across all training conditions. We did not have these images rated for valence, but note that past studies have used these images as neutral controls, demonstrating a differential impact to emotional images (e.g., Most et al., 2005; Onie and Most, 2017; Jin et al., 2018). Finally, in our study, we specifically instructed individuals to ignore the images appearing before the probe, but in previous studies participants tended not to be explicitly instructed to ignore the images (e.g., Basanovic et al., 2017; Clarke et al., 2017). Therefore, it is possible that instructions to explicitly ignore these images led participants to exert attentional control in a way that reduced the impact of the emotional images themselves. This may be a more likely possibility among healthy participants (as in our study) than among highly anxious individuals, who have previously exhibited deficits in attentional control (Eysenck et al., 2007; Eysenck and Derakshan, 2011). It is possible that this contributed to our finding that traditional ABM training conditions did not modify attention. That is, the images themselves may not have captured attention in the first place, resulting in no consequence of the contingency manipulation.

Furthermore, one issue previously noted is that studies have found the Dot Probe to have low reliability, which may have contributed to previous null findings. Although the reliability of the attentional bias indices in our study (0.50–0.53) are higher than those reported elsewhere in the literature, they are still lower than the recommended 0.7 cut-off (Nunnally, 1978), potentially affecting our findings. In this study, we used McDonald’s Omega to compute reliability instead of Cronbach’s Alpha, which is commonly used in the attentional bias literature. We opted to use McDonald’s Omega, as Cronbach’s alpha has shown to be less accurate in the face of violated assumptions, whilst the Omega is less so (Zinbarg et al., 2005). In our case, Cronbach’s Alpha almost always overestimates the reliability value.

Despite the fact that the Dot Probe has been heavily criticized for its lack of reliability, a recent study found that reliability may not be the best way to evaluate the usefulness of a task. Reliability indices can be heavily affected by between participant-variability, and many cognitive tasks with reliable effects may yield low reliability (Hedge et al., 2018). The authors of that study noted that any calculation designed to reduce noise, e.g., obtaining a bias index by subtracting two values, would almost always reduce between-participant variability, and therefore reliability. Consistent with this, one issue is that not all ABM studies report reliability indices, including those that have found training effects. Therefore, at this time, it is difficult to evaluate the role of reliability in the current context.

In addition to the traditional ABM training conditions, the inhibition control training also did not modify attentional bias. One possibility is it that inhibition training, even if successful, does not causally influence attentional bias. In one study, Heeren et al. (2015) did find that a two session ABM training program improved executive control as measured by the Attention Network Task (Fan et al., 2005), but it may be that this causal relationship is unidirectional. ABM training may improve attentional control, consistent with hypotheses by Chen et al. (2015) and McNally (2018), but attentional training may not modify attentional bias. This is a possibility that should be addressed by future research, which would also benefit from delineating between spatial attentional control and executive control, as Heeren et al. (2015) found that ABM modified executive control, but not spatial attentional control. In addition, future studies could also benefit from having pre and post measures of inhibitory control to see whether manipulations of inhibitory control were successful or not.

One potential issue is that traditional attentional retraining conditions have two stimuli on the screen during training whilst the current inhibitory control training condition had only one. Thus, it could be argued that this stimulus-level difference impedes easy comparison across the training conditions. However, attentional training in the ‘inhibitory control’ condition is based on the well-validated anti-cueing task, which involves reorienting away from a pre-potent, attention-grabbing stimulus. Furthermore, the presence of one- versus two- stimuli in the training conditions does not account for our failure to observe a difference between the attend- and avoid- negative groups.

An additional avenue for future research might be to assess whether inhibitory control can improve performance in non-spatial indices of attentional bias, such as emotion-induced blindness (Most et al., 2005), which has been shown to have higher test-retest reliability than typically seen with the dot probe (Onie and Most, 2017). It will also be informative to investigate whether inhibitory control mediates the effect of training on attentional bias by assessing attentional control (Basanovic et al., 2017) after training but prior to the assessment of attentional bias.

Importantly, we used Bayesian inference to analyze the robustness of the attentional bias assessment data that was obtained after attentional bias modification. Both the frequentist and Bayesian approach indicated no training effects when using the full sample. However, the Bayesian approach also distinguished between a lack of an effect and a lack of power, with the resulting Bayes factors suggesting strong evidence to support the null. This was further bolstered when we intentionally reduced power by reducing the sample size to match the sample size used in previous studies, in which we found that over 1000 iterations, the frequentist analysis yielded a 1.6% Type I error, and potentially 98.4% type II error due to low power. The Bayesian approach yielded a 0.1% Type I error, and 0.1% Type-II error, whilst suggesting there was either only anecdotal or moderate evidence 99.8% of the time. In the frequentist analysis, a non-significant *p* value was unable to indicate whether there was true lack of an effect, or a lack of precision. However, the Bayes Factor was sensitive to how much evidence was collected, therefore indicating a measure of precision as well. This demonstrates one of the strengths of the Bayesian approach, which quantifies evidence rather than utilizing a specific cut-off. Note that in this analysis we did use a cut-off of BF = 10 to aid in comparing the two approaches; however, in practice, the Bayes Factor is used to quantify evidence, e.g., we would be able to make stronger conclusions with a Bayes Factor of 9 than a Bayes Factor of 3.5, despite falling into the same bracket in Jeffrey’s scale. An interesting finding is that the frequentist approach to this analysis only yielded 1.6% Type-I error, successfully controlling for error at α = 0.05. Therefore, our results seem to suggest that the frequentist approach is not invalid in and of itself in controlling for Type I error. However, this analysis method may prove difficult in controlling for Type II error. Power analyses have been used to reduce these concerns; however, sample size estimates from power analyses rely on effect size estimates from past findings, which may be subject to a number of issues such as publication bias or simply random differences in samples.

It is worth noting that JASP is designed as an introduction to Bayesian statistics, and therefore still has limited functionality, e.g., a lack of a graphical representation of the posterior distributions. Therefore, we highly encourage readers to pursue further Bayesian approaches using programs such as JAGS and winBUGS. There are several great resources and books that discuss these topics (e.g., Kruschke, 2015).

In conclusion, we set out to compare the outcome of inhibition training with traditional ABM training conditions and found that inhibition training appeared to have no impact on attentional bias. However, the interpretability of this manipulation is limited due to the lack of change in attentional bias in the more traditional attentional bias training conditions as well. Nevertheless, we are able to provide strong evidence against any ABM training effects in the current sample of healthy participants, made possible using Bayesian analyses. To our knowledge this is the first instance where evidence has been provided against the presence of this effect (as opposed to simply failing to find the effect). These findings highlight the need for greater understanding of the mechanisms underlying ABM. Bayesian analysis approaches may prove to be useful tools in this quest due to their ability to distinguish between a lack of power and lack of an effect.

## Data Availability Statement

The datasets for this study can be found in the open science framework, https://osf.io/hfr9s/.

## Author Contributions

SO and SBM designed the experiments. SO conducted the experiments and performed the analyses. All authors contributed to the final manuscript.

## Conflict of Interest Statement

The authors declare that the research was conducted in the absence of any commercial or financial relationships that could be construed as a potential conflict of interest.

## References

[B1] Aczel B. Palfi B. Szollosi A. Kovacs M. Szaszi B. Szecsi P. (2018). Quantifying support for the null hypothesis in psychology: an empirical investigation. *Adv. Methods Pract. Psychol. Sci.* 1 357–366. 10.1177/2515245918773742

[B2] Andrews M. Baguley T. (2013). Prior approval: the growth of Bayesian methods in psychology. *Br. J. Math. Stat. Psychol.* 66 1–7. 10.1111/bmsp.12004 23330865

[B3] Bar-Haim Y. Lamy D. Pergamin L. Bakermans-Kranenburg M. van IJzendoorn M. (2007). Threat-related attentional bias in anxious and nonanxious individuals: a meta-analytic study. *Psychol. Bull.* 133 1–24. 10.1037/0033-2909.133.1.1 17201568

[B4] Basanovic J. Notebaert L. Grafton B. Hirsch C. Clarke P. (2017). Attentional control predicts change in bias in response to attentional bias modification. *Behav. Res. Ther.* 99 47–56. 10.1016/j.brat.2017.09.002 28917715

[B5] Browning M. Holmes E. A. Murphy S. E. Goodwin G. M. Harmer C. J. (2010). Lateral prefrontal cortex mediates the cognitive modification of attentional bias. *Biol. Psychiatry* 67 919–925. 10.1016/j.biopsych.2009.10.031 20034617 PMC2866253

[B6] Button K. Ioannidis J. Mokrysz C. Nosek B. Flint J. Robinson E. (2013). Power failure: why small sample size undermines the reliability of neuroscience. *Nat. Rev. Neurosci.* 14 365–376. 10.1038/nrn3475 23571845

[B7] Cain M. Prinzmetal W. Shimamura A. Landau A. (2014). Improved control of exogenous attention in action video game players. *Front. Psychol.* 5:69. 10.3389/fpsyg.2014.00069 24575061 PMC3918660

[B8] Chen N. Clarke P. Watson T. MacLeod C. Guastella A. (2015). Attentional bias modification facilitates attentional control mechanisms: evidence from eye tracking. *Biol. Psychol.* 104 139–146. 10.1016/j.biopsycho.2014.12.002 25527400

[B9] Clarke P. Browning M. Hammond G. Notebaert L. MacLeod C. (2014). The causal role of the dorsolateral prefrontal cortex in the modification of attentional bias: evidence from transcranial direct current stimulation. *Biol. Psychiatry* 76 946–952. 10.1016/j.biopsych.2014.03.003 24690113

[B10] Clarke P. J. F. Branson S. Chen N. T. M. Van Bockstaele B. Salemink E. MacLeod C. (2017). Attention bias modification training under working memory load increases the magnitude of change in attentional bias. *J. Behav. Ther. Exp. Psychiatry* 57 25–31. 10.1016/j.jbtep.2017.02.003 28257926

[B11] Derakshan N. D. Ansari T. L. Hansard M. Shoker L. Eysenck M. W. (2009). Anxiety, inhibition, efficiency, and effectiveness: an investigation using the antisaccade task. *Exp. Psychol.* 56 48–55. 10.1027/1618-3169.56.1.48 19261578

[B12] Everaert J. Mogoaşe C. David D. Koster E. (2015). Attention bias modification via single-session dot-probe training: failures to replicate. *J. Behav. Ther. Exp. Psychiatry* 49 5–12. 10.1016/j.jbtep.2014.10.011 25468204

[B13] Eysenck M. (1992). *Anxiety.* Hove: Lawrence erlbaum.

[B14] Eysenck M. Derakshan N. (2011). New perspectives in attentional control theory. *Pers. Individ. Dif.* 50 955–960. 10.1111/j.1467-8624.2012.01738.x 22364371

[B15] Eysenck M. Derakshan N. Santos R. Calvo M. (2007). Anxiety and cognitive performance: attentional control theory. *Emotion* 7 336–353. 10.1037/1528-3542.7.2.336 17516812

[B16] Fan J. McCandliss B. D. Fossella J. Flombaum J. I. Posner M. I. (2005). The activation of attentional networks. *NeuroImage* 26 471–479. 10.1016/j.neuroimage.2005.02.00 15907304

[B17] Grafton B. Ang C. MacLeod C. (2012). Always look on the bright side of life: the attentional basis of positive affectivity. *Eur. J. Pers.* 26 133–144. 10.1002/per.1842

[B18] Grafton B. Mackintosh B. Vujic T. MacLeod C. (2014). When ignorance is bliss: explicit instruction and the efficacy of CBM-A for anxiety. *Cogn. Ther. Res.* 38 172–188. 10.1007/s10608-013-9579-3

[B19] Hedge C. Powell G. Sumner P. (2018). The reliability paradox: why robust cognitive tasks do no produce reliable individual differences. *Behav. Res. Methods* 50 1166–1186. 10.3758/s13428-017-0935-1 28726177 PMC5990556

[B20] Heeren A. De Raedt R. Koster E. H. W. Philippot P. (2013). The (neuro)cognitive mechanisms behind attention bias modification in anxiety: proposals based on theoretical accounts of attentional bias. *Front. Hum. Neurosci.* 7:119. 10.3389/fnhum.2013.00119 23576969 PMC3616236

[B21] Heeren A. Mogoaşe C. McNally R. J. Schmitz A. Philippot P. (2015). Does attention bias modification improve attentional control? A double-blind randomized experiment with individuals with social anxiety disorder. *J. Anxiety Disord.* 29 35–42. 10.1016/j.janxdis.2014.10.007 25465885

[B22] Iacoviello B. M. Wu G. Abend R. Murrough J. W. Feder A. Fruchter E. (2014). Attention bias variability and symptoms of posttraumatic stress disorder. *J. Trauma. Stress* 27 232–239. 10.1002/jts.21899 24604631 PMC4617532

[B23] Jeffreys H. (1961). *Theory of Probability* 3rd Edn. Oxford: Clarendon Press.

[B24] Jin D. Onie S. Curby K. Most S. (2018). Aversive images cause less perceptual interference among violent video game players: evidence from emotion-induced blindness. *Vis. Cogn.* [Preprint]. 10.1080/13506285.2018.1553223

[B25] Kanyongo G. Brook G. Kyei-Blankson L. Gocmen G. (2007). Reliability and statistical power: how measurement fallibility affects power and required sample sizes for several parametric and nonparametric statistics. *J. Mod. Appl. Stat. Methods* 6 81–90. 10.22237/jmasm/1177992480

[B26] Kruijt A. Field A. Fox E. (2018). Capturing dynamics of biased attention: are new attention variability measures the way forward? *PLoS One* 11:e0166600. 10.1371/journal.pone.0166600 27875536 PMC5119769

[B27] Kruschke J. K. (2015). *Doing Bayesian Data Analysis: A Tutorial with R, JAGS and Stan* 2nd Edn. Cambridge, MA: Academic Press.

[B28] Lang P. J. Bradley M. M. Cuthbert B. N. (1997). *International Affective Picture System (IAPS): Technical Manual and Affective Ratings.* Gainesville, FL: NIMH Center for the Study of Emotion and Attention.

[B29] Lovibond P. Lovibond S. (1995). The structure of negative emotional states: comparison of the Depression Anxiety Stress Scales (DASS) with the beck depression and anxiety inventories. *Behav. Res. Ther.* 33 335–343. 10.1016/0005-7967(94)00075-U 7726811

[B30] MacLeod C. Clarke P. (2015). The attentional bias modification approach to anxiety intervention. *Clin. Psychol. Sci.* 3 58–78. 10.1177/2167702614560749

[B31] MacLeod C. Mathews A. Tata P. (1986). Attentional bias in emotional disorders. *J. Abnorm. Psychol.* 95 15–20. 10.1037/0021-843X.95.1.15 3700842

[B32] Mathews A. MacLeod C. (2002). Induced processing biases have causal effects on anxiety. *Cogn. Emot.* 16 331–354. 10.1080/02699930143000518

[B33] McNally R. (2018). Attentional bias for threat: crisis or opportunity? *Clin. Psychol. Rev.* 10.1016/j.cpr.2018.05.005 [Epub ahead of print]. 29853421

[B34] Milkins B. Notebaert L. MacLeod C. Clarke P. (2016). The potential benefits of targeted attentional bias modification on cognitive arousal and sleep quality in worry-related sleep disturbance. *Clin. Psychol. Sci.* 4 1015–1027. 10.1177/2167702615626898

[B35] Most S. Chun M. Widders D. Zald D. (2005). Attentional rubbernecking: cognitive control and personality in emotion-induced blindness. *Psychon. Bull. Rev.* 12 654–661. 10.3758/BF03196754 16447378

[B36] Notebaert L. Clarke P. Grafton B. MacLeod C. (2015). Validation of a novel attentional bias modification task: the future may be in the cards. *Behav. Res. Ther.* 65 93–100. 10.1016/j.brat.2014.12.007 25594940

[B37] Nunnally J. C. (1978). *Psychometric Theory* 2nd Edn. New York, NY: McGraw-Hill.

[B38] Onie S. Most S. (2017). Two roads diverged: distinct mechanisms of attentional bias differentially predict negative affect and persistent negative thought. *Emotion* 17 884–894. 10.1037/emo0000280 28230392

[B39] Posner M. I. Cohen Y. Rafal R. D. (1982). Neural systems control of spatial orienting. *Philos. Trans. R. Soc. Lond. Ser. B Biol. Sci.* 298 187–198. 10.1098/rstb.1982.0081 6125970

[B40] Price R. B. Kuckertz J. M. Siegle G. J. Ladouceur C. D. Silk J. S. Ryan N. D. (2015). Empirical recommendations for improving the stability of the dot-probe task in clinical research. *Psychol. Assess.* 27 365–376. 10.1037/pas0000036 25419646 PMC4442069

[B41] Price R. B. Wallace M. Kuckertz J. M. Amir N. Graur S. Cummings L. (2016). Pooled patient-level meta-analysis of children and adults completing a computer-based anxiety intervention targeting attentional bias. *Clin. Psychol. Rev.* 50 37–49. 10.1016/j.cpr.2016.09.009 27693664 PMC5118070

[B42] Rouder J. Morey R. (2012). Default bayes factors for model selection in regression. *Multivariate Behav. Res.* 47 877–903. 10.1080/00273171.2012.734737 26735007

[B43] Schmukle S. (2005). Unreliability of the dot probe task. *Eur. J. Pers.* 19 595–605. 10.1002/per.554

[B44] Theeuwes J. (1992). Perceptual selectivity for color and form. *Percept. Psychophys.* 51 599–606. 10.3758/BF03211656 1620571

[B45] Van Bockstaele B. Verschuere B. Tibboel H. De Houwer J. Crombez G. Koster E. H. (2014). A review of current evidence for the causal impact of attentional bias on fear and anxiety. *Psychol. Bull.* 140 682–721. 10.1037/a0034834 24188418

[B46] Wagenmakers E. J. Beek T. F. Rotteveel M. Gierholz A. Matzke D. Steingroever H., (2015). Turning the hands of time again: a purely confirmatory replication study and a bayesian analysis. *Front. Psychol.* 6:494. 10.3389/fpsyg.2015.00494 25964771 PMC4408755

[B47] Wieser M. Pauli P. Mühlberger A. (2009). Probing the attentional control theory in social anxiety: an emotional saccade task. *Cogn. Affect. Behav. Neurosci.* 9 314–322. 10.3758/CABN.9.3.314 19679766

[B48] Zinbarg R. E. Revelle W. Yovel I. Li W. (2005). Cronbach’s α, Revelle’s β, and McDonald’s ωH : their relations with each other and two alternative conceptualizations of reliability. *Psychometrika* 70 123–133. 10.1007/s11336-003-0974-7

